# Difficult Airway Management in a Conscious Patient With Ankylosing Spondylitis: A Case Report

**DOI:** 10.7759/cureus.104596

**Published:** 2026-03-03

**Authors:** Luisa Carvalho, Joana Santos, Lúcia Gonçalves, Elisabete Valente

**Affiliations:** 1 Anesthesiology, Unidade Local de Saúde da Região de Leiria, Leiria, PRT

**Keywords:** airway management, ankylosing spondylitis, endotracheal intubation, laryngoscopy, procedural sedation

## Abstract

Ankylosing spondylitis (AS) is a chronic inflammatory disease that may cause cervical deformity and ankylosis, complicating airway management. The success rate of direct laryngoscopy in this population is low, and awake endotracheal intubation with minimal cervical manipulation is recommended. We report the case of a 65-year-old man with AS and complete loss of cervical mobility scheduled for laparoscopic cholecystectomy. Airway evaluation revealed Mallampati III, mouth opening >3 cm, sternomental distance of 10 cm, and severe restriction of cervical extension. Due to the unavailability of equipment for awake fibreoptic intubation (AFI), videolaryngoscopy under remifentanil sedation and topical lidocaine was chosen. Orotracheal intubation (C-MAC D-Blade) was successfully performed, followed by general anaesthesia without complications. The patient was safely extubated after the procedure. This case suggests awake videolaryngoscopy as an alternative for airway management in patients with AS when fibreoptic bronchoscopy is not feasible.

## Introduction

Ankylosing spondylitis (AS) is a chronic rheumatologic inflammatory disease that affects the axial skeleton and surrounding tissues [1]. The condition shows a marked male predominance, with men approximately three times more commonly affected than women. Progressive fusion of the spinal elements results in loss of flexibility of the back and neck (the classic “bamboo spine”). Cervical involvement is frequent, occurring in up to 70% of patients after two decades of disease progression [2].

Airway management in AS is challenging. Patients frequently demonstrate reduced atlanto-occipital extension, which can be assessed clinically by measuring cervical extension, sternomental distance, and fixed flexion deformity. These parameters are incorporated into structured airway assessment tools, such as the LEMON criteria [3]. Patients may also present with reduced mouth opening and intolerance of the supine position due to fixed anterior flexion of the cervical spine [2]. These pathological changes render the spine vulnerable to injury even from low-energy forces, including those generated during direct laryngoscopy [4]. A fused, anteriorly displaced cervical spine fixed in flexion, often with ossified supporting ligaments, may not safely tolerate a chin lift, jaw thrust, or the laryngoscopy required to pass an endotracheal tube [4]. Severe neurological injury, including permanent tetraplegia, has been reported after direct laryngoscopy in this population, and neck extension may precipitate vertebrobasilar insufficiency through bony encroachment on the vertebral artery [4]. Because direct laryngoscopy in AS is associated with low first-pass success and higher complication rates, it should be avoided whenever possible [4].

A meticulous preoperative history and examination are therefore essential. Beyond documenting disease duration and current therapies, clinicians should assess functional status and degree of disability using validated instruments such as the Bath Ankylosing Spondylitis Functional Index (BASFI) [5] and objectively evaluate cervical spine mobility by measuring range of motion, particularly flexion and extension. Structured airway assessment frameworks (e.g., LEMON approach) may assist in systematic identification of difficult airway predictors [3]. As many patients cannot tolerate the supine position, additional cervical support may be required.

Traditionally, awake fibreoptic intubation (AFI) has been considered the reference technique in severe AS because it preserves spontaneous ventilation and minimises neck motion [6]. However, it is a complex technique requiring regular practice and skill to be performed successfully. Videolaryngoscopy has been reported as an alternative to AFI, requiring simpler equipment and a shorter learning curve, with successful outcomes reported in patients with difficult airways [6,7]. Awake nasal intubation with videolaryngoscopy has also been described as a feasible approach in selected patients [8].

## Case presentation

**Figure 1:**
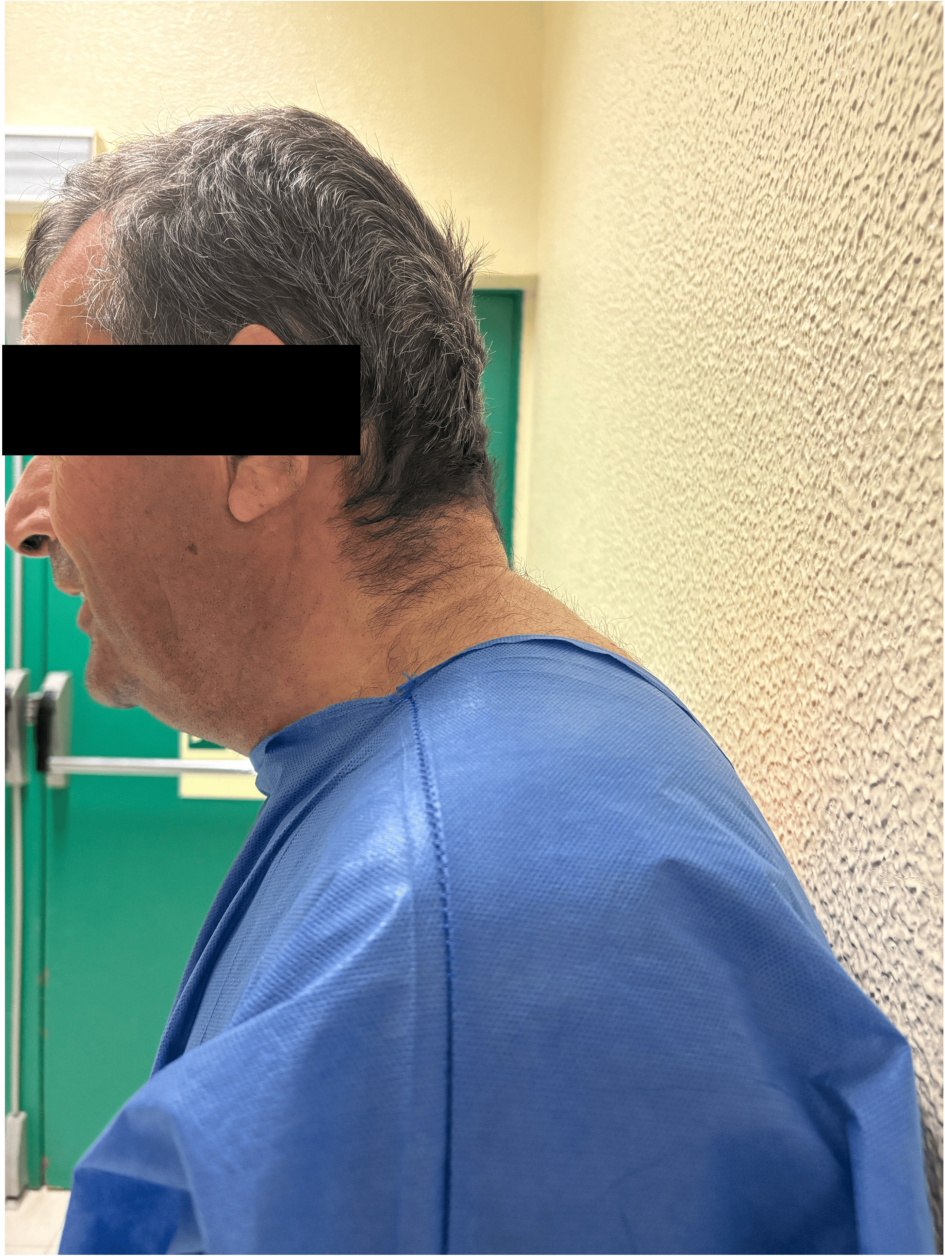
Marked limitation of cervical extension Fixed cervical flexion deformity consistent with advanced ankylosing spondylitis. No measurable active or passive atlanto-occipital extension beyond the patient’s baseline flexed posture was achievable on clinical examination.

**Figure 2:**
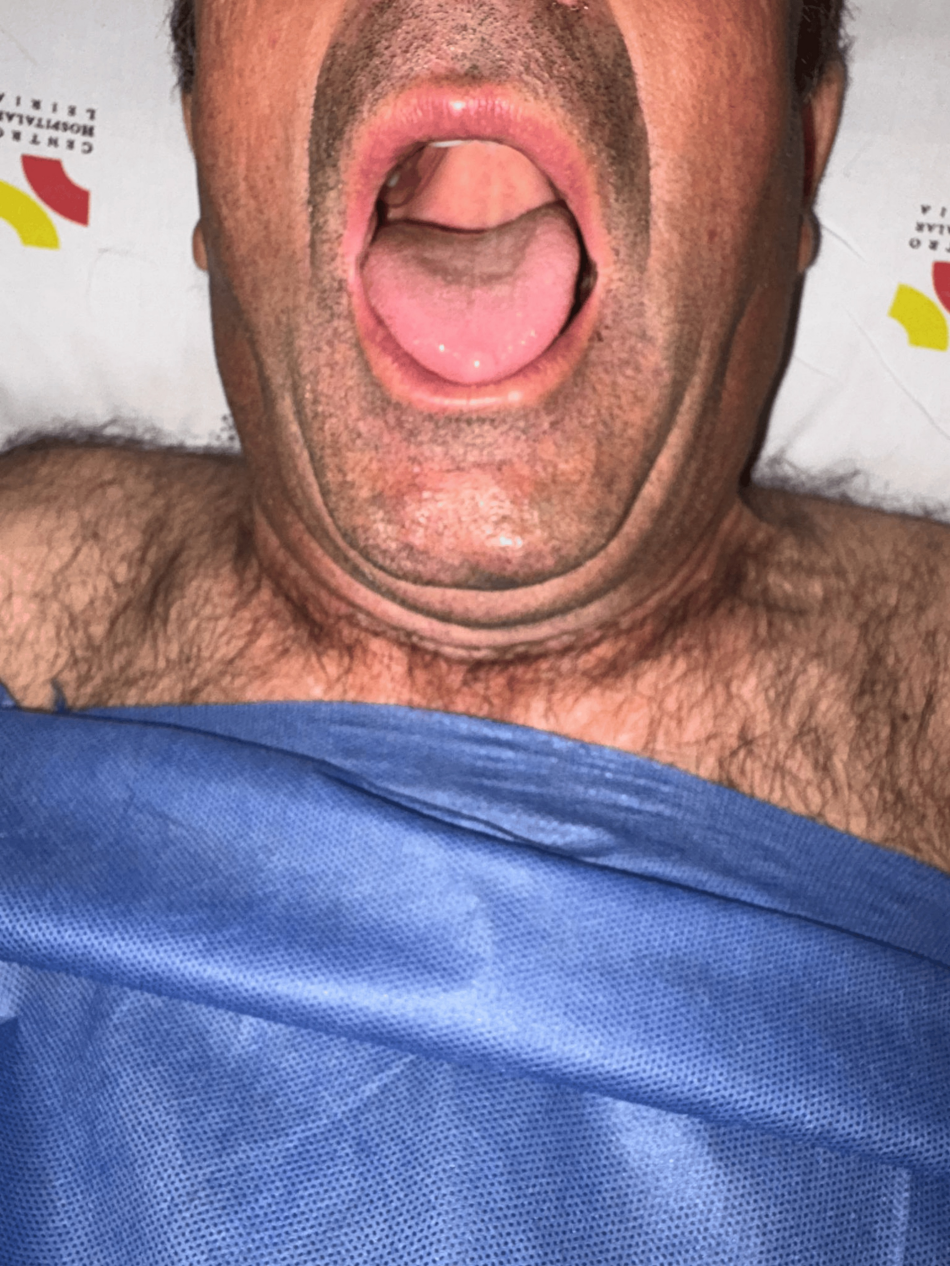
Mallampati III and mouth opening >3 cm

We describe a 65-year-old man (ASA III) with a >10-year history of AS who was electively scheduled for laparoscopic cholecystectomy for cholelithiasis and gallbladder polyps. He was a former smoker and reported no known drug allergies. His surgical history included a total thyroidectomy for a toxic multinodular goitre 10 years before, with no airway complications on record. Preoperative airway assessment was performed according to the LEMON approach [3]. External inspection revealed no craniofacial abnormalities and no clinical signs of upper airway obstruction. Evaluation demonstrated Mallampati class III, inter-incisor distance of 3 cm, thyromental distance of 6 cm, sternomental distance of 10 cm, and severely restricted cervical range of motion with inability to achieve atlanto-occipital extension (Figure 1), consistent with fixed flexion deformity. Mouth opening was greater than 3 cm (Figure 2). Given the history of previous cervical surgery, the potential difficulty of emergency front-of-neck access due to altered anatomy and scar tissue formation was carefully considered. A structured, stepwise airway management plan was formulated. Awake videolaryngoscopy was selected as the primary technique, with supraglottic airway devices immediately available as secondary options. In anticipation of a failed intubation scenario, equipment for emergency front-of-neck access (including scalpel, bougie, and tracheal tube) was prepared and checked prior to airway instrumentation. The surgical team was informed, and readiness for prompt cricothyrotomy was ensured.

The patient required additional cervical support to tolerate the supine position. Routine preoperative blood tests, electrocardiogram, and chest radiography were unremarkable. Given the risk of a difficult airway, awake tracheal intubation under sedation and topical anaesthesia was proposed and discussed as a safer strategy than attempting airway management after induction of general anaesthesia. A flexible bronchoscope was unavailable, which prompted the choice of videolaryngoscopy.

In the operating room, standard ASA monitoring was applied. This patient was able to tolerate the supine position without respiratory compromise when adequate cervical support was provided. Two cushions were positioned to maintain the patient’s baseline neutral alignment and minimise cervical movement. A single 20-G peripheral intravenous cannula was inserted. Supplemental oxygen was administered throughout the procedure via nasal cannula, maintaining spontaneous ventilation. Preoxygenation was performed prior to airway instrumentation using 100% oxygen delivered via facemask to maximise oxygen reserves. Bag-mask ventilation equipment was immediately available in the event of desaturation or need for rapid sequence induction. Sedation was achieved with a titrated remifentanil infusion (up to 0.1 mcg/kg/min). Topicalisation consisted of 10% lidocaine spray applied intranasally and in the oropharynx (total volume of 4 mL); no additional nerve blocks were performed. The patient remained cooperative, and first-attempt orotracheal intubation was successfully performed using videolaryngoscopy (C-MAC, D-Blade) with a 7.0-mm ID cuffed endotracheal tube, yielding a near-complete glottic view (estimated POGO score of 90-100%). Correct placement was confirmed by visualisation of the tube passing the vocal cords, a normal capnographic waveform, and bilateral chest auscultation. General anaesthesia was then induced with propofol 2 mg/kg, lidocaine 1 mg/kg, and rocuronium 0.6 mg/kg. Anaesthesia was maintained with sevoflurane, and the patient was ventilated in a controlled mode. Surgery lasted one hour and 40 minutes and was uneventful. The patient was successfully extubated in the immediate postoperative period. Metamizole (2 g) and paracetamol (1 g) were administered for postoperative analgesia.

## Discussion

AS predominantly involves the axial skeleton and can lead to progressive ankylosis and kyphotic deformity. Cervical spine disease restricts head and neck mobility and increases the risk of neurological or vascular injury with airway manipulation [4]. Techniques that minimise cervical movement are therefore essential.

Predictors of a difficult airway include limited mouth opening, high Mallampati class, and markedly reduced cervical extension. Additional recognised risk factors include obesity, short or thick neck, reduced thyromental distance, limited sternomental distance, restricted mandibular protrusion, and a history of previous difficult intubation [2]. In this patient, severely restricted cervical mobility and a high Mallampati class were the most prominent predictors. A thorough preoperative assessment (documenting functional limitations, the ability to tolerate the supine position, and a stepwise airway plan with rescue options) is mandatory. In our case, a structured backup plan was established prior to airway manipulation. Rescue strategies included readiness for bag-mask ventilation, availability of a second-generation supraglottic airway device, and immediate preparation for emergency front-of-neck access in a “cannot intubate, cannot oxygenate” scenario. All necessary equipment and experienced personnel were present in the operating room.

If AFI is not feasible, videolaryngoscopy alone or in combination with flexible bronchoscopy (“double visualisation”) has been used successfully [9]. Regardless of the device chosen, success depends on effective topical anaesthesia, titration of sedation (e.g., remifentanil or dexmedetomidine), patient-specific positioning (semi-upright, ramped), and strict maintenance of neutral cervical alignment. A robust backup strategy should be immediately available, including readiness for front-of-neck access as the final option if non-invasive methods fail [10-12].

## Conclusions

Severe cervical involvement in AS makes airway management difficult and potentially hazardous. This case shows that awake videolaryngoscopic intubation, supported by careful topical anaesthesia, titrated sedation, patient-specific positioning, and strict neutral cervical alignment, can secure the airway safely and predictably. The strategy preserved spontaneous ventilation until the tube was in place and avoided the neck movement required for direct laryngoscopy. Although AFI remains the reference technique, awake videolaryngoscopy is a practical alternative in selected patients when an experienced operator and appropriate equipment are available.

Good outcomes rely on thorough preoperative assessment, clear communication with the patient, team preparedness, and a stepwise plan with immediately accessible rescue options. Simple measures such as additional cervical support, generous topicalisation, and cautious sedation meaningfully enhance safety and comfort. This case supports prioritising an awake, minimal-movement strategy in AS with fixed cervical deformity. Wider adoption of standardised institutional pathways and regular team rehearsal (including front-of-neck access readiness) may further reduce complications in this high-risk population.
